# High aldehyde dehydrogenase activity at diagnosis predicts relapse in patients with t(8;21) acute myeloid leukemia

**DOI:** 10.1002/cam4.2422

**Published:** 2019-07-30

**Authors:** Lu Yang, Wen‐Min Chen, Feng‐Ting Dao, Yan‐Huan Zhang, Ya‐Zhe Wang, Yan Chang, Yan‐Rong Liu, Qian Jiang, Xiao‐Hui Zhang, Kai‐Yan Liu, Xiao‐Jun Huang, Ya‐Zhen Qin

**Affiliations:** ^1^ Peking University People's Hospital Peking University Institute of Hematology, National Clinical Research Center for Hematologic Disease Beijing China

**Keywords:** aldehyde dehydrogenase, flow cytometry, minimal residual disease, relapse, RUNX1‐RUNX1T1, t(8;21) acute myeloid leukemia

## Abstract

Acute myeloid leukemia (AML) with t(8;21) is a heterogeneous disease. Although the detection of minimal residual disease (MRD), which is indicated by RUNX1‐RUNX1T1 transcript levels, plays a key role in directing treatment, risk stratification needs to be improved, and other markers need to be assessed. A total of 66 t(8;21) AML patients were tested for aldehyde dehydrogenase (ALDH) activity by flow cytometry at diagnosis, and 52 patients were followed up for a median of 20 (1‐34) months. The median percentage of CD34+ALDH+, CD34+CD38‐ALDH+, and CD34+CD38+ALDH+ cells among nucleated cells were 0.028%, 0.012%, and 0.0070%, respectively. The CD34+ALDH+‐H, CD34+CD38‐ALDH+‐H, and CD34+CD38+ALDH+‐H statuses (the percentage of cells that were higher than the individual cutoffs) were all significantly associated with a lower 2‐year relapse‐free survival (RFS) rate in both the whole cohort and adult patients (*P* = .015, .016, and .049; *P* = .014, .018, and .032). Patients with < 3‐log reduction in the RUNX1‐RUNX1T1 transcript level after the second consolidation therapy (defined as MRD‐H) had a significantly lower 2‐year RFS rate than patients with ≥ 3‐log reduction (MRD‐L) (*P* = .017). The CD34+ALDH+ status at diagnosis was then combined with the MRD status. CD34+ALDH+‐L/MRD‐H patients had similar 2‐year RFS rates to both CD34+ALDH+‐L/MRD‐L and CD34+ALDH+‐H/MRD‐L patients (*P* = .50 and 1.0); and CD34+ALDH+‐H/MRD‐H patients had significantly lower 2‐year RFS rate compared with CD34+ALDH+‐L and/or MRD‐L patients (*P* < .0001). Multivariate analysis showed that CD34+ALDH+‐H/MRD‐H was an independent adverse prognostic factor for relapse. In conclusion, ALDH status at diagnosis may improve MRD‐based risk stratification in t(8;21) AML, and concurrent high levels of CD34+ALDH+ at diagnosis and MRD predict relapse.

## Graphical abstract

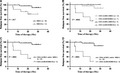

## 1 INTRODUCTION

Although t(8;21) acute myeloid leukemia (AML) is considered to have a good prognosis, relapse occurs in up to 40% of patients treated with chemotherapy.^1^, ^2^, ^3^, ^4^, ^5^, ^6^ Therefore, stratification is needed in order to guide appropriate treatment. Minimal residual disease (MRD) levels indicated by RUNX1‐RUNX1T1 transcript levels as well as c‐KIT mutations have been demonstrated to be strong prognostic factors in t(8;21) AML.^4^, ^5^, ^6^, ^7^, ^8^, ^9^ However, their risk predictions are not perfect, and other markers have yet to be evaluated.

Leukemia stem cells (LSCs) may cause relapse from the complete remission (CR) state.^10^ CD34+CD38‐ is a putative immunophenotype of LSCs with the ability to generate leukemia in immunodeficient mice.^11^, ^12^, ^13^ However, this immunophenotype has been challenged because some studies have demonstrated that LSCs might exist in CD34+CD38+ and CD34‐ cells.^14^, ^15^, ^16^ Thus, this surface immunophenotype alone might be inadequate for identifying LSCs.

Aldehyde dehydrogenases (ALDHs) are a family of cytosolic enzymes that are involved in various biological processes.^17^, ^18^ Cells with high ALDH activity (ALDH+) have been identified as cancer stem cells in various solid tumors, including breast, lung, and ovarian cancer.^19^, ^20^, ^21^ Furthermore, it has been demonstrated that ALDH alone or in combination with CD34 can be used to purify hematopoietic stem cells.^22^, ^23^ In AML, some studies have shown that LSCs might be enriched among the ALDH+ subsets.^22^, ^24^, ^25^ A high percentage of ALDH+ was demonstrated to be associated with adverse cytogenetic factors.^24^, ^25^, ^26^, ^27^ Whether the percentage of ALDH+ is prognostic within the same cytogenetic risk group, such as t(8;21), is unknown. In addition, a clinical cohort study in intermediate and high cytogenetic risk AML showed that patients with a high percentage of ALDH + had adverse outcomes.^24^, ^26^, ^27^ However, this effect has not been evaluated in t(8;21) AML patients to date.

In the present study, we examined ALDH activity in 66 t(8;21) AML patients at diagnosis and evaluated its sole prognostic role and the impact of its combination with MRD on relapse.

## 2 MATERIAL AND METHODS

### 2.1 Patients and treatment

A total of 66 t(8;21) AML patients were enrolled in the present study. These patients were diagnosed at our hospital from September 2015 to July 2018. A t(8;21) AML diagnosis was determined according to morphologic evaluation of bone marrow (BM) smears, immunophenotyping, cytogenetics, and molecular analyses. In total, 44 (66.7%) patients were male. The median age of the patients at diagnosis was 38 (range: 2‐66) years. Patients younger than and older than 14 years at diagnosis were categorized as pediatric and adult patients, respectively.

Overall, 52 patients received treatment and were followed up at our hospital. As we previously reported, induction therapy consisted of 1‐2 cycles of the “3 + 7” regimen or the homoharringtonine, aclarubicin, and cytarabine regimen for the 37 adult patients, and cytarabine, idarubicin, and etoposide were used for the 15 pediatric patients.^6^, ^7^, ^28^ Forty‐eight patients received consolidation therapy after achieving CR. Among them, 36 received an intermediate‐dose cytarabine‐based chemotherapy only, and 12 received chemotherapy followed by allogeneic hematopoietic stem cell transplantation (allo‐HSCT) from a human leukocyte antigen (HLA)‐identical sibling (n = 9) or an HLA haplotype‐matched relative (n = 3) in CR1. The allo‐HSCT conditioning regimen and graft‐versus‐host disease prophylaxis have been described previously.^29^ Dasatinib was used in four patients with c‐KIT mutations when the reduction of the RUNX1‐RUNX1T1 transcript levels was less than 3‐log after the second cycle of consolidation. The cutoff date for follow‐up was October 2018. This study was approved by the Ethics Committee of Peking University People's Hospital. All patients provided written informed consent in accordance with the Declaration of Helsinki to participate in the present study.

### 2.2 Flow cytometry analysis

Bone marrow (BM) was aspirated from all 66 patients at diagnosis. Red blood cells were lysed using ammonium chloride solution (STEMCELL Technologies, Vancouver, Canada). ALDH activity of nucleated cells was detected using an ALDEFLUOR Kit (STEMCELL Technologies), according to the manufacturer's instructions. Nucleated cells of each sample were adjusted to a concentration of 1 × 10^6^/mL. The cells were incubated with the ALDH‐substrate BODIPY‐aminoacetaldehyde with or without the ALDH inhibitor diethylaminobenzaldehyde (DEAB). Test and control cells were then incubated at 37°C for 30 minutes, centrifuged, and resuspended in ice‐cold ALDEFLUOR buffer. Cells were then incubated with fluorochrome‐labeled mouse anti‐human monoclonal antibodies for 30 minutes on ice and then resuspended in ALDEFLUOR buffer. Testing and data acquisition were performed using a FACS Canto II (Becton Dickinson, San Jose CA, USA). Analysis was performed using Kaluza flow analysis software (Beckman Coulter, Brea, CA, USA).

The monoclonal antibodies that were used included CD45‐PerCP, CD34‐PE‐Cy7, CD38‐APC, and Lineage‐APC‐H7/Cy7 (consisting of CD3, CD14, CD16, CD20, and CD36). CD16, CD20, and CD36 antibodies were purchased from BioLegend (San Diego, CA, USA), and the others were purchased from BD Biosciences (San Jose, CA, USA).

**Figure 1:**
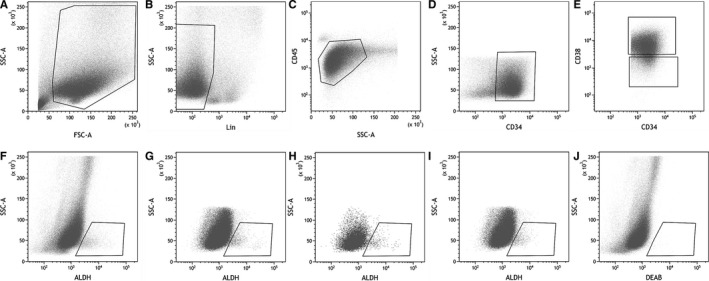
Gating strategy for flow cytometric testing of ALDH+, CD34+ALDH+, CD34+CD38‐ALDH +, and CD34+CD38+ALDH+ cells. Sequential gating is shown from A to E. (A) nucleated cells; (B) Lin‐ cells; (C) blast cells; (D) CD34+ cells; (E) CD34+CD38‐ and CD34+CD38+ cells. (F) ALDH+; (G) CD34+ALDH+; (H) CD34+CD38‐ALDH+; (I) CD34+CD38+ALDH+; (J) negative control

The gating strategy is shown in Figure 1. An forward scattering/side scattering (FSC/SSC) plot was used to exclude cellular debris and nonviable cells (Figure 1A). Lymphocytes, monocytes, macrophages, natural killer cells, and erythrocytes were excluded by lineage‐negative (Lin‐) (Figure 1B), blast cells were defined as SSClow/CD45dim (Figure 1C), CD34+ was defined in CD34/SSC plot (Figure 1D), and CD34+CD38‐ and CD34+CD38+ were derived from CD34+ cells (Figure 1E). Cells with DEAB‐sensitive ALDH activity were defined as ALDH+ (Figure 1F‐I).

### 2.3 Detection of RUNX1‐RUNX1T1 transcript levels and c‐KIT mutation and MRD monitoring

RNA extraction, complementary DNA (cDNA) synthesis, and TaqMan‐based real‐time quantitative PCR technology were used as described previously.^6^ The RUNX1‐RUNX1T1 transcript level was calculated as the percentage of RUNX1‐RUNX1T1 transcript copies/ABL copies. The pretreatment baseline level of RUNX1‐RUNX1T1 transcripts was 388% in our laboratory. According to our previous reports, a high MRD level (MRD‐H) was defined as less than a 3‐log reduction in RUNX1‐RUNX1T1 transcript level compared to baseline after the second cycle of consolidation chemotherapy (>0.4%).^6^

As we previously reported, cDNA was used to perform PCR to test for c‐KIT mutations in exons 17 and 8.^7^, ^8^

### 2.4 Statistical analyses and definitions

Pairwise comparisons of the variables between groups were performed using the Fisher's exact test for categorical variables. A receiver operating characteristic (ROC) curve was used to identify the optimal cutoff levels that best discriminated patients with relapse. Survival functions were estimated using the Kaplan‐Meier method and were compared using the log‐rank test. Relapse‐free survival (RFS) was measured from the date when CR was achieved to relapse. Overall survival (OS) was measured from diagnosis to death (regardless of the cause) or patients were queried at the date of last follow‐up to determine whether they were still alive, or were censored on the date they were last known to be alive. Variables associated with *P* < .2 in the univariate analysis were entered into a multivariate analysis performed by the Cox models. The level for a statistically significant difference was set at *P* < .05. The SPSS 19.0 software package (SPSS Inc), and GraphPad Prism 5 (GraphPad Software Inc) were used for data analysis.

## 3 RESULTS

### 3.1 Patient characteristics and outcomes

The characteristics of all patients at diagnosis are summarized in Table 1. A total of 27 (40.9%) patients had c‐KIT mutations. In the 39 patients who were followed up until the second consolidation therapy, 11 (28.2%) had < 3‐log reduction in the RUNX1‐RUNX1T1 transcript level compared to baseline (>0.4%) and were defined as MRD‐H, and the other 28 patients had ≥ 3‐log reduction and were defined as MRD‐L.^6^

**Table 1:** Characteristics of the patients at diagnosis

Variables	Value
N	66
Age (y, median; range)	38 (2‐66)
Sex (male/female)	44/22
White blood cell (WBC) count (×10^9^/L; median; range)	8.8 (1.7‐58.2)
Hemoglobin (g/L; median; range)	81 (42‐126)
Platelet count (×10^9^/L; median; range)	32.5 (2‐312)
Blasts in BM (%, median; range)	51 (15‐89)
Patients with cytogenetic abnormalities other than t(8;21) (n = 62)	41 (66.1%)
RUNX1‐RUNX1T1 transcript level	555.0% (123.7%‐1880.5%)
Patients with c‐KIT mutations	27 (40.9%)

A total of 52 patients were followed up for a median of 20 (1‐34) months. About 50 (96.2%) patients achieved CR after 1‐3 cycles of induction therapy, and seven (14.0%) of these patients relapsed, all of whom received chemotherapy only. The 2‐year RFS rate of the 50 patients who achieved CR was 78.3% (95% confidence interval (CI) 57.9%‐89.6%). Three (5.8%) of 52 patients died during follow‐up, and the 2‐year OS rate of the 52 patients was 92.6% (95%CI 78.3%‐97.6%).

### 3.2 The percentage of ALDH+cells in t(8;21) AML patients at diagnosis

Of all 66 patients, the median percentage of ALDH+cells among nucleated cells was 0.17% (range 0.0029%‐6.8%), and that were 0.028% (range 0.0013%‐3.0%), 0.012% (range 0.00028%‐0.62%), and 0.0070% (range 0%‐2.1%) for CD34+ALDH+, CD34+CD38‐ALDH+, and CD34+CD38+ALDH+ cells among nucleated cells, respectively.

### 3.3 Determination of the optimal cutoff values for patient grouping

The ROC curve analysis was performed in 48 follow‐up patients who achieved CR and received consolidation therapy. Although the percentages of ALDH+, CD34+ALDH+, CD34+CD38‐ALDH+, and CD34+CD38+ALDH+ cells could not significantly differentiate patients who relapsed (*P* = .23, .065, .072, and .088), trends existed, and 0.34%, 0.065%, 0.024%, and 0.0094% were determined as the individual optimal cutoff values according to the Youden index.

We referred to patients with percentages of ALDH+, CD34+ALDH+, CD34+CD38‐ALDH+, and CD34+CD38+ALDH+ cells that were higher than the cutoff values as ALDH+‐H, CD34+ALDH+‐H, CD34+CD38‐ALDH+‐H, and CD34+CD38+ALDH+‐H, and the patients with percentages of cells that were lower than the cutoff values as ALDH+‐L, CD34+ALDH+‐L, CD34+CD38‐ALDH+‐L, and CD34+CD38+ALDH+‐L. Thus, among the 48 follow‐up patients, 13 (27.1%), 15 (31.3%), 16 (33.3%), and 23 (47.9%) patients were categorized as ALDH+‐H, CD34+ALDH+‐H, CD34+CD38‐ALDH+‐H, and CD34+CD38+ALDH+‐H, respectively.

### 3.4 Impact of the percentage of ALDH+cells on the achievement of CR

In the entire cohort, the CR rate after the first course of induction was 76.9% (40/52). The percentages of ALDH+, CD34+ALDH+, CD34+CD38‐ALDH+, and CD34+CD38+ALDH+ cells all had no impact on the achievement of CR (H group vs L group: 92.3% vs 71.8%, *P* = .25; 73.3% vs 78.4%, *P* = .98; 76.5% vs 77.1%, *P* = 1.0; 70.8% vs 82.1%, *P* = .34).

### 3.5 Impact of the percentage of ALDH+ cells on relapse

**Figure 2:**
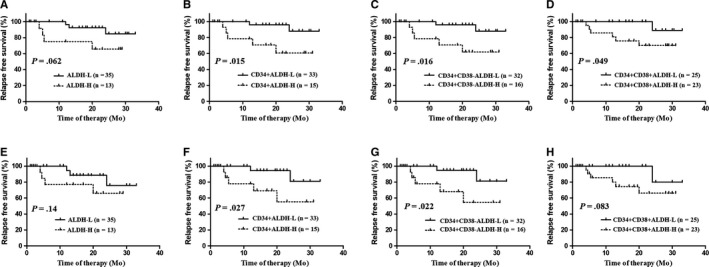
RFS analysis of the 48 follow‐up patients based on ALDH+grouping. (A‐D) no censoring; (E‐H) censoring at the time of allo‐HSCT; (A, E) ALDH+; (B, F) CD34+ALDH+; (C, G) CD34+CD38‐ALDH+; (D, H) CD34+CD38+ALDH+

In the 48 follow‐up patients who achieved CR and received consolidation therapy, ALDH+‐H patients tended to have a lower 2‐year RFS rate than ALDH+‐L patients (65.6% [95% CI 32.0%‐85.6%] vs 84.7% [95% CI 57.0%‐95.2%], *P* = .062, Figure 2A). However, CD34+ALDH+‐H patients had a significantly lower 2‐year RFS rate compared to CD34+ALDH+‐L patients (60.6% [95% CI 28.7%‐81.8%] vs 88.0% [95% CI 57.2%‐97.1%], *P* = .015, Figure 2B). Furthermore, both CD34+CD38‐ALDH+‐H and CD34+CD38+ALDH+‐H patients had a significantly lower 2‐year RFS rate compared to CD34+CD38‐ALDH+‐L and CD34+CD38+ALDH+‐L patients, respectively (61.9% [95% CI 30.7%‐82.3%] vs 88.0% [95% CI 57.2%‐97.1%], *P* = .016, Figure 2C; 70.1% [95% CI 44.8%‐85.4%] vs 88.9% [95% CI 43.3%‐98.4%], *P* = .049, Figure 2D).

Next, the 12 patients who underwent allo‐HSCT were censored at the time of transplantation. ALDH+‐H patients had a similar 2‐year RFS rate to ALDH+‐L patients (64.3% [95% CI 29.8%‐85.1%] vs 76.4% [95% CI 38.3%‐92.7%], *P* = .14, Figure 2E). However, CD34+ALDH+‐H and CD34+CD38‐ALDH+‐H patients were both individually significantly related to a lower 2‐year RFS rate compared with that of CD34+ALDH+‐L and CD34+CD38‐ALDH+‐L patients (55.4% [95% CI 21.2%‐79.9%] vs 81.0% [95% CI 37.8%‐95.5%], *P* = .027, Figure 2F; 54.5% [95% CI 20.4%‐79.4%] vs 81.2% [95% CI 37.9%‐95.6%], *P* = .022, Figure 2G). In addition, CD34+CD38+ALDH+‐H patients tended to have a lower 2‐year RFS rate than CD34+CD38+ALDH+‐L patients (66.1% [95% CI 38.2%‐83.7%] vs 80.0% [95% CI 20.4%‐96.9%], *P* = .083, Figure 2H).

Thirty‐three adult patients who achieved CR and received consolidation therapy were further analyzed. As shown in Figure S1, ALDH+‐H and CD34+ALDH+‐H patients individually had significantly lower 2‐year RFS rate compared with ALDH+‐L and CD34+ALDH+‐L patients (*P* = .035, Figure S1A; *P* = .014, Figure S1B). Furthermore, both CD34+CD38‐ALDH+‐H and CD34+CD38+ALDH+‐H patients had significantly lower 2‐year RFS rates compared with CD34+CD38‐ALDH+‐L and CD34+CD38+ALDH+‐L patients, respectively (*P* = .018, Figure S1C; *P* = .032, Figure S1D). Similar results existed if the 11 patients who underwent allo‐HSCT were censored at the time of transplantation (Figure S1E‐H).

Because the percentages of both CD34+CD38‐ALDH+ and CD34+CD38+ALDH+ had an impact on relapse, the CD34+ALDH+ subset was used in the subsequent analysis.

### 3.6 Univariate analysis of variables other than the percentage of ALDH+ cells

**Figure 3:**
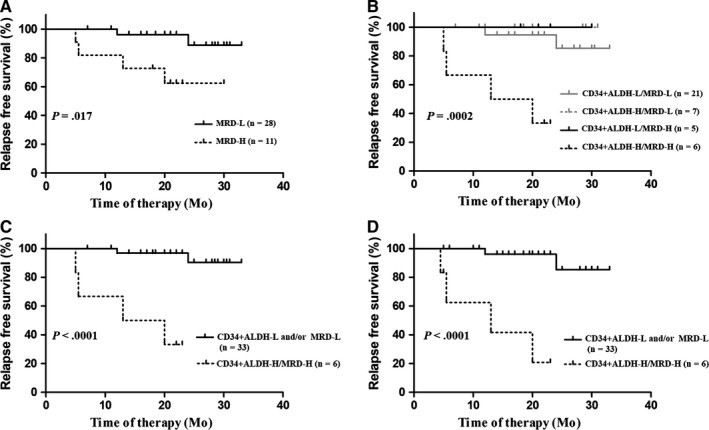
RFS of patients grouped by MRD (A); combination of CD34+ALDH+ and MRD (B); CD34+ALDH+‐L and/or MRD‐L and CD34+ALDH+‐H/MRD‐H (C); CD34+ALDH+‐L and/or MRD‐L and CD34+ALDH+‐H/MRD‐H with censoring at the time of allo‐HSCT (D)

Among 39 patients who were followed up at least until the second course of consolidation therapy, MRD‐H patients had a significantly lower 2‐year RFS rate than MRD‐L patients (62.3% [95% CI 27.7%‐84.0%] vs 88.8% [95% CI 59.7%‐97.3%], *P* = .017, Table 2, Figure 3A), and pediatric patients had a significantly lower 2‐year RFS rate than adult patients (*P* = .032). In addition, treatment with chemotherapy only tended to be significantly related to a lower 2‐year RFS rate compared with that of treatment with allo‐HSCT (*P* = .070). In contrast, white blood cell (WBC) count, hemoglobin, platelet count, blast percentage in BM, cytogenetic abnormalities, other than t(8;21), CR achievement after the first induction therapy, and c‐KIT mutation status at diagnosis had no impact on the 2‐year RFS rate (all *P* > .05).

**Table 2:** Univariate analysis of relapse in the follow‐up patients (n = 48)

Variables	No. of patients	2‐year RFS rate (95% CI)	HR (95% CI)	*P* value
Age				
Pediatric patients	15	100%	1.0	.032
Adult patients	33	65.3% (37.4%‐83.2%)	5.3 (1.2‐24.3)	
Sex				
Male	31	88.7% (69.0%‐96.2%)	1.0	.17
Female	17	57.3% (20.1%‐82.4%)	3.0 (0.62‐14.6)	
WBC count at diagnosis				
≤ 10 × 10^9^/L	28	85.9% (61.9%‐95.3%)	1.0	.33
> 10 × 10^9^/L	20	67.9% (31.1%‐87.9%)	2.1 (0.47‐9.7)	
Hemoglobin				
≤ 80 g/L	24	80.2% (48.5%‐93.5%)	1.0	.64
> 80 g/L	24	78.6% (51.5%‐91.6%)	1.4 (0.32‐6.4)	
Platelet count				
≤ 35 × 10^9^/L	23	81.9% (53.8%‐93.8%)	1.0	.94
> 35 × 10^9^/L	25	74.2% (41.2%‐90.4%)	0.95 (0.21‐4.3)	
Blasts in BM				
≤ 50%	24	71.0% (36.3%‐89.0%)	1.0	.79
> 50%	24	85.0% (60.4%‐94.9%)	0.82 (0.18‐3.6)	
Patients with cytogenetic abnormalities other than t(8;21)				
No	16	75.2% (40.7%‐91.4%)	1.0	.45
Yes	30	78.4% (49.3‐92.0)	0.53 (0.10‐2.7)	
c‐KIT gene				
Wild‐type	29	71.2% (44.6%‐86.7%)	1.0	.25
Mutation	19	93.3% (61.2%‐99.0%)	0.40 (0.085‐1.9)	
Treatment modality				
Allo‐HSCT	12	100%	1.0	.070
Chemotherapy only	36	68.9% (42.4%‐85.1%)	4.4 (0.89‐21.5)	
CR after the first induction therapy				
Yes	40	76.2% (52.3%‐89.3%)	1.0	.78
No	8	87.5% (38.7%‐98.1%)	0.76 (0.11‐5.2)	
MRD status				
MRD‐L	28	88.8% (59.7%‐97.3%)	1.0	.017
MRD‐H	11	62.3% (27.7%‐84.0%)	9.7 (1.5‐63.0)	

### 3.7 The impact of the combination of the percentage of CD34+ALDH+ cells with MRD on relapse

By combining the percentage of CD34+ALDH+ cells with MRD levels, 39 patients were categorized into the following four groups: CD34+ALDH+‐L/MRD‐L (n = 21), CD34+ALDH+‐H/MRD‐L (n = 7), CD34+ALDH+‐L/MRD‐H (n = 5), and CD34+ALDH+‐H/MRD‐H (n = 6).

As shown in Figure 3B, the 2‐year RFS rate was significantly different among the four groups (*P* = .0002). In MRD‐H patients, CD34+ALDH+‐H/MRD‐H patients had a significantly lower 2‐year RFS rate than CD34+ALDH+‐L/MRD‐H patients (33.3% [95% CI 4.6%‐67.6%] vs 100%, *P* = .036). Furthermore, CD34+ALDH+‐L/MRD‐H patients had a similar 2‐year RFS rate to both CD34+ALDH+‐L/MRD‐L and CD34+ALDH+‐H/MRD‐L patients (100% vs 85.3%, *P* = .50; 100% vs 100%, *P = *1.0). Therefore, these three groups were merged into the CD34+ALDH+‐L and/or MRD‐L group. As a result, CD34+ALDH+‐H/MRD‐H patients had a significantly lower 2‐year RFS rate compared to CD34+ALDH+‐L and/or MRD‐L patients (33.3% [95% CI 4.6%‐67.6%] vs 90.3% [95% CI 64.2%‐97.7%], *P* < .0001, Figure 3C). Similar results were observed if the patients who underwent allo‐HSCT were censored at the time of transplantation (20.8% [95% CI 0.87%‐59.5%] vs 85.3% [95% CI 47.7%‐96.7%], *P* < .0001, Figure 3D).

Of 11 MRD‐H patients, four patients relapsed. After considering the percentage of CD34+ALDH+ cells along with MRD, five patients were placed into the CD34+ALDH‐L/MRD‐H group and none of these patients relapsed; all four of the relapsed patients were placed into the CD34+ALDH‐H/MRD‐H group. Therefore, ALDH may improve MRD‐based risk stratification in t(8;21) AML.

### 3.8 CD34+ALDH+‐H/MRD‐H status independently predicts relapse

CD34+ALDH+‐H/MRD‐H status, age, sex, and treatment modality were all used in a multivariate analysis of 39 patients who were followed up at least until the second course of consolidation therapy. The results showed that CD34+ALDH+‐H/MRD‐H status was the only independent adverse prognostic factor for relapse (HR 27.5 [95% CI 3.1‐246.4], *P* = .0030).

## 4 DISCUSSION

AML with t(8;21) is a heterogeneous disease, and other prognostic factors need to be assessed in addition to MRD.^2^ In the present study, ALDH activity was evaluated in 66 t(8;21) AML patients at diagnosis. We found that high percentages of CD34+ALDH+, CD34+CD38‐ALDH +, and CD34 + CD38+ALDH + cells as well as high MRD levels were significantly related to relapse. After combining the percentage of CD34+ALDH+ at diagnosis with MRD, patients were regrouped, and CD34+ALDH+‐H/MRD‐H status was an independent adverse prognostic factor for relapse in t(8;21) AML.

Over the past decade, molecular markers have been incorporated into the risk stratification system in t(8;21) AML. Several studies, including ours, have demonstrated that high MRD levels, which are indicated by less than a 3‐log reduction of RUNX1‐RUNX1T1 transcript levels during treatment, were poor prognostic factors, although the significant time points were different among the studies.^4^, ^5^, ^6^ In addition, c‐KIT mutation was found to be the most prevalent mutation in t(8;21) AML and has been demonstrated to be related to poor outcomes by many studies.^7^, ^8^, ^9^ However, the specificity and sensitivity of the current risk stratification are not perfect. Whether other parameters could complement the use of molecular markers needs to be evaluated in t(8;21) AML.

Relapse was a major challenge for the long‐term outcome of AML patients and may be caused by the persistence of LSCs. LSCs have been reported to be quiescent, well protected within the bone marrow niche, resistant to chemotherapy, and responsible for recurrent disease.^30^ Some reports have demonstrated that the frequency of LSCs in patients with AML might be prognostic.^31^, ^32^ Evidence for the identification of LSCs involves a demonstration of the capacity to successfully reconstitute leukemia by engrafting cells into immunodeficient mice.^11^, ^12^, ^13^ However, mouse engraftment could not be used in clinical practice due to its operational complexity. Thus, it is necessary to find feasible methods to identify LSCs and evaluate their clinical significance.

ALDHs are an NAD(P)^+^‐dependent enzyme superfamily that are involved in various biological processes.^17^, ^18^ Many reports have demonstrated that ALDH is expressed at high levels in cancer stem cells.^19^, ^20^, ^21^, ^25^, ^26^, ^27^ In AML, ALDH was found to be highly expressed in LSCs and to be related to chemotherapy resistance.^22^, ^24^, ^25^, ^26^, ^27^

Several studies have evaluated the prognostic role of ALDH in AML. Cheung et al reported that in patients, a high percentage of ALDH+ cells was associated with adverse cytogenetic abnormalities.^25^ Ran et al demonstrated that AML patients with a higher percentage of ALDH+ cells had a high number of genetic and molecular risk factors and poor clinical outcomes.^24^, ^26^ Hoang et al analyzed intermediate‐risk AML and found that patients with a high percentage of ALDH+ cells had shorter disease‐free survival and OS than patients with a low percentage of ALDH+ cells.^27^ In a prospective clinical study, Gerber et al reported that patients with a high percentage of CD34+CD38‐ALDH + cells manifested a significantly lower CR rate and lower event‐free and OS rates.^33^ A total of five t(8;21) AML and 13 AML patients with favorable cytogenetic risk were included in the above five studies, and all of them were categorized as ALDH+‐L.^24^, ^25^, ^26^, ^27^, ^33^ It implied that among AML, patients with t(8;21) usually had low percentage of ALDH+, which is in consistent with their low cytogenetic risk. In the present study, similar prognostic significance of the percentage of ALDH+ was seen within t(8;21) AML; that is, a high percentage of CD34 + ALDH+ cells was correlated with a low RFS rate.

Since MRD is the most important prognostic molecular marker in t(8;21) AML, which was similarly demonstrated in the current study, we combined the ALDH with MRD parameters. We found that in patients, both CD34+CD38‐ALDH+‐H and CD34+CD38+ALDH+‐H status had an adverse impact on the 2‐year RFS rate. CD34+CD38‐ is widely accepted as an immunophenotype of LSCs, but CD34+CD38+ cells were also found to have LSC characteristics.^14^ Pearce et al reported that the CD34+ and ALDH+ subpopulations in AML largely overlapped, and CD34+ALDH+ cells possess the CD34+CD38‐ and CD34+CD38+ phenotypes.^22^ Therefore, we did not consider CD38 expression, and we combined the CD34+ALDH+ and MRD parameters to categorize patients into four groups. The combined results showed that only patients with concurrent high levels of CD34+ALDH+ at diagnosis and MRD had a high relapse risk. Furthermore, CD34+ALDH+‐H/MRD‐H was found to be the only independent adverse prognostic factor. Our results demonstrated the usefulness of ALDH for improving MRD‐based risk stratification in t(8;21) AML.

In conclusion, ALDH may improve MRD‐based risk stratification in t(8;21) AML, and concurrent high levels of CD34+ALDH+ at diagnosis and MRD predict relapse. Although this is a retrospective study and the sample size was no large enough, it gave us a clue to the more precise stratification in t(8;21) AML. Prospective studies with larger sample sizes are warranted.

## CONFLICT OF INTEREST

The authors declare no conflict of interest.

## AUTHOR CONTRIBUTIONS

LY wrote manuscript; WMC performed the PCR analysis; LY, FTD, YHZ YZW, and YC performed flow cytometry; QJ, XHZ, KYL, and XJH collected clinical data; YZQ designed the study and revised the manuscript. All authors gave final approval.
