# Supplementary figures and images for: High aldehyde dehydrogenase activity at diagnosis predicts relapse in patients with t(8;21) acute myeloid leukemia
Source: Cancer Med. 2019 Jul 30;8(12):5459–67. doi: 10.1002/cam4.2422 (PMC6745853; doi:10.1002/cam4.2422)

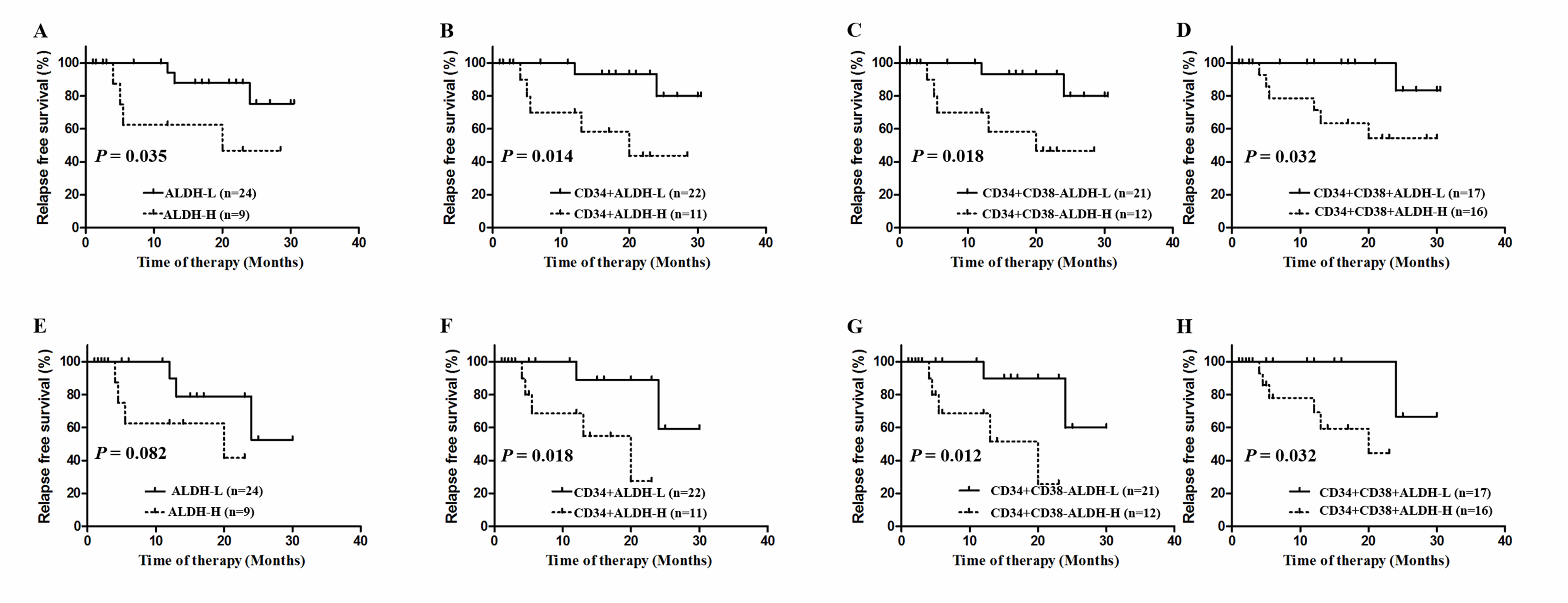

Supplement: Supplementary file 1 [file CAM4-8-5459-s001.tif]
